# In Silico Exploration of Foods and Compounds That Modulate Nicotine Metabolism

**DOI:** 10.3390/nu18193162

**Published:** 2026-09-25

**Authors:** Marilena M. Bourdakou, Elena Philippou, Iosif Orfanos, George M. Spyrou, Lefteris C. Zacharia

**Affiliations:** 1 Bioinformatics Department, The Cyprus Institute of Neurology and Genetics, 2371 Nicosia, Cyprus; 2 Department of Life Sciences, School of Life and Health Sciences, University of Nicosia, 2417 Nicosia, Cyprus; 3 Department of Health Sciences, School of Life and Health Sciences, University of Nicosia, 2417 Nicosia, Cyprus; 4 Bioactive Molecules Research Center, School of Life and Health Sciences, University of Nicosia, 2417 Nicosia, Cyprus

**Keywords:** nicotine metabolism, CYP2A6, natural inhibitors, foods, dietary combinations

## Abstract

**Background/Objectives**: Targeting nicotine metabolism through inhibition of cytochrome P450 2A6 (CYP2A6) has been proposed as a potential strategy for modulating nicotine exposure. CYP2A6 metabolizes nicotine and several clinically relevant compounds, while genetic polymorphisms cause inter-individual variability in nicotine metabolism. Natural products represent accessible and cost-effective sources of compounds with potential relevance to CYP2A6-mediated metabolism. This study aimed to identify naturally occurring food-derived compounds with potential relevance to nicotine metabolism using a combined computational and experimental approach. **Methods**: A three-step forward/backward workflow was applied. First, CYP2A6-related foods and associated bioactive compounds were identified using FooDisNET and majority voting analysis. Second, selected compounds were experimentally evaluated for inhibitory activity against CYP2A5, the functional orthologue of human CYP2A6, using a murine liver microsome assay. Third, validated inhibitory compounds were traced back to foods and dietary patterns potentially capable of modulating nicotine metabolism. **Results**: Experimental validation identified four compounds with inhibitory activity against CYP2A5: geraniol, benzaldehyde, 1,8-cineole, and camphor. These compounds were associated with foods including Chinese cinnamon, Mandarin orange (Clementine, Tangerine), Turmeric, Fennel, Corn, Ginger, *Artemisia montana*, *Artemisia capillaris*, *Akebia trifoliata*, *Mosla chinensis*, *Akebia quinata*, *Elsholtzia ciliata*, *Artemisia princeps*, and *Artemisia argyi*. **Conclusions**: These findings identify food-derived compounds with experimentally demonstrated CYP2A5 inhibitory activity and potential relevance to CYP2A6-mediated nicotine metabolism, supporting their further investigation as nutrition-based approaches to modulating nicotine metabolism and smoking reduction or cessation.

## 1. Introduction

Tobacco smoking poses a substantial global public health risk, with approximately 1.3 billion people currently using tobacco products worldwide. Eurostat data indicate that in Europe, 19.7% of the population smokes daily. Smoking has been linked to cardiovascular disease, respiratory disorders, various cancers, and other serious health conditions [1]. The risks associated with smoking are in part tied to nicotine addiction, which is influenced by the rate at which nicotine is metabolized in the body. Individuals who metabolize nicotine more rapidly often experience stronger addictive behaviors, as they require higher and more frequent doses to achieve the same effects, thereby reinforcing dependency [2].

Targeting nicotine metabolism, particularly through inhibiting the cytochrome P450 2A6 (CYP2A6) enzyme, has emerged as a promising strategy to combat nicotine dependence [3] CYP2A6 plays a crucial role in metabolizing nicotine and several other clinically relevant substances. However, due to genetic polymorphisms, there is considerable variability in CYP2A6 activity among individuals, resulting in different nicotine metabolism rates [4]. Naturally derived products present a valuable and accessible source of CYP2A6 inhibitors, especially beneficial for their wide availability, cost-effectiveness, and ease of extraction compared to synthetic compounds [5]. These natural inhibitors often display fewer side effects and are typically well-tolerated in the body, making them appealing candidates for long-term use in smoking cessation strategies. Additionally, natural compounds may offer multifunctional benefits, such as anti-inflammatory and antioxidant properties, which could further support overall health during smoking cessation [6].

Previous efforts to identify CYP2A6 inhibitors have largely focused on individual natural compounds or specific medicinal plants, primarily using in vitro assays or targeted screening approaches. For example, several flavonoids, including apigenin, tangeretin, galangin, and isorhamnetin, have been reported to inhibit CYP2A6 activity, while active constituents from medicinal plants such as *Cyanthillium cinereum* (formerly *Vernonia cinerea*) and cinnamon-derived compounds, including trans-cinnamaldehyde and 2-methoxycinnamaldehyde, have also demonstrated inhibitory effects on CYP2A6 [5,7,8,9]. While these studies have provided valuable insights into CYP2A6 inhibition, they typically do not establish a systematic connection between bioactive compounds and their broader dietary sources, thereby limiting their translational relevance for nutritional or lifestyle-based interventions.

More broadly, computational approaches have been employed to identify dietary bioactive compounds with predicted inhibitory activity against clinically relevant enzymes and to prioritize candidates for experimental testing. For instance, virtual screening strategies supported by machine-learning models have been applied to discover dietary inhibitors of the drug-metabolizing enzyme CYP3A4 from food- and supplement-derived compound libraries, followed by experimental validation of selected predictions [10]. Despite these advances, comparable computational strategies have not been systematically applied to CYP2A6, the primary enzyme responsible for nicotine metabolism, particularly in the context of identifying naturally occurring, food-derived inhibitors.

This study aimed to identify naturally occurring compounds and food sources with potential relevance to CYP2A6-mediated nicotine metabolism. By integrating computational analysis with experimental validation, we sought to highlight accessible, diet-derived candidates that may support strategies for reducing nicotine dependence and facilitating smoking cessation.

## 2. Methods

In order to identify natural compounds and foods with the potential to inhibit CYP2A6-mediated nicotine metabolism, a combined computational and experimental workflow was employed. First, a computational analysis was performed to identify CYP2A6-related foods and their associated bioactive compounds using the FooDisNET database (https://hisbim.life.nctu.edu.tw/FooDisNET/) [11] (accessed on 4 April 2024). Subsequently, selected compounds were experimentally evaluated for their inhibitory activity using a liver microsome-based assay employing murine CYP2A5, the functional orthologue of human CYP2A6. Finally, foods containing compounds experimentally validated for CYP2A5 inhibition were investigated to explore their potential relevance within dietary patterns associated with nicotine metabolism modulation.

More specifically, a three-step approach was used: First, a computational analysis was performed to identify CYP2A6-related foods and their associated bioactive compounds. Second, selected compounds were experimentally evaluated for their inhibitory activity using a liver microsome-based assay, employing the murine CYP2A5 enzyme as the functional orthologue of human CYP2A6 [12]. Third, CYP2A6-related foods containing all the compounds experimentally validated for CYP2A5 inhibition were investigated. The potential dietary combinations of these foods that could lead to nicotine metabolism mediation are discussed.

### 2.1. Identification of CYP2A6-Related Foods and Compounds

A search for CYP2A6-related foods was undertaken using the FooDisNET database, a resource designed to map associations between foods, bioactive compounds, protein targets, and disorders. At the time of access (June 2024), FooDisNET contained 6329 foods, 53,920 compounds, and 22,865 target proteins, of which 4092 were linked to 18,689 disorders. For each CYP2A6-related food, the number of associated compounds retrieved through FooDisNET was determined and used as a database-based criterion for food prioritization. Subsequently, all compounds associated with the CYP2A6-related foods were investigated. These compounds were considered candidates for further analysis and prioritization. A majority voting analysis was performed to identify the compounds most frequently associated with CYP2A6-related foods. This analysis enabled us to determine which compounds are present across multiple CYP2A6-related foods, thereby highlighting those with the highest prevalence.

### 2.2. Experimental Validation

#### 2.2.1. Microsome Preparation

Microsomes containing the murine CYP2A5 enzyme, the functional orthologue of human CYP2A6, were prepared from C57Bl/6 mouse liver using the precipitation technique. Briefly, mice were euthanized by cervical dislocation and the livers quickly excised. Livers were washed with ice-cold phosphate-buffered saline (PBS), blotted, dried, weighted and then minced. The liver pool was then homogenized on ice in a polytetrafluoroethylene (PTFE) pestle in a glass homogenizer by adding 3.5 mL extraction buffer (10 mM Hepes pH 7.8, 250 mM sucrose, 25 mM potassium chloride, 1 mM EGTA) for each gram of tissue. Homogenate was then centrifuged at 1000× *g* for 10 min at 4 °C; the supernatant was removed and centrifuged at 12,000× *g* for 15 min at 4 °C. The new supernatant was then transferred into a tube on ice and calcium chloride (8 mM) equal to 7.5 volumes of the supernatant was slowly added with stirring. The resulting sample was then centrifuged at 8000× *g* for 10 min, and the pellet was resuspended in extraction buffer (0.3 mL per gr original tissue). Protein content was determined by the Bradford assay.

#### 2.2.2. Inhibition Studies

The biochemical reaction consisted of MgCl_2_, coumarin (10 μM, concentration that gives half-maximal response), microsomes (45 μg), NADPH (1 mM), phosphate buffer to a final volume of 200 μL in the presence or absence of the test compounds (0.05 to 1 mM final concentrations). The reaction was started by the addition of NADPH (1 mM), 5 min after pre-incubation with the substrate. The reaction mixture was then incubated in a water bath set at 37 °C for 30 min. Immediately after, the tubes were placed on ice and the reaction was quenched by the addition of 150 μL of Tris-HCl pH 9. One hundred (100) μL of the resulting solution were transferred to a black fluorescent plate and measured at excitation/emission of 355/460 nm with an automatic plate reader (Victor X3, Perkin Elmer, Springfield, IL, USA) for detection of the fluorescent coumarin metabolite 7-hydroxycoumarin. Results are presented relative to the control (without test compound).

#### 2.2.3. Statistics

Statistical analysis was performed using a one-way ANOVA, comparison of means, with Tukey’s multiple comparison test for comparison between groups. IC_50_ was calculated using GraphPad Prism 8.0.2 by Nonlinear regression of log (inhibitor) vs. response—Variable slope (four parameters), and reported with its 95% confidence interval.

## 3. Results

### 3.1. CYP2A6-Related Foods and Compounds: Key Findings and Insights

The FooDisNET database search identified a total of 3078 foods associated with CYP2A6 (Supplementary File S1). Table 1 presents the top 20 CYP2A6-related foods, ranked based on the number of related compounds. A higher number of associated compounds indicates a greater number of FooDisNET-derived associations between the food and CYP2A6.

The investigation of all compounds associated with the CYP2A6-related foods identified 196 unique compounds, which are provided in Supplementary File S2. These compounds were considered candidates for further prioritization and investigation as potential modulators of CYP2A6-related metabolism.

### 3.2. Majority Voting Analysis of Compounds Associated with CYP2A6-Related Foods

A majority voting analysis was performed to identify the compounds most frequently associated with CYP2A6-related foods. This analysis allowed the determination of the compounds that appear across multiple CYP2A6-related foods, highlighting those with the highest prevalence. Figure 1 presents the top 20 compounds, ranked by the number of CYP2A6-related foods in which they are involved. As presented in Figure 1, Nicotinic acid ranked highest, being present in 803 CYP2A6-related foods, followed by alpha-Tocopherol (661 foods) and L-Ascorbic acid (604 foods). Glycine was found in 586 foods, while trans-Ferulic acid and Citric acid were associated with 373 and 317 foods, respectively. Other notable compounds include Eugenol (257 foods), Benzoic acid (211 foods), and Geraniol (195 foods). Compounds such as Vitamin D (194 foods), Benzaldehyde (183 foods), and (E)-Resveratrol (168 foods) were also highly prevalent. Chrysin and 1,8-cineole appeared in 164 and 152 foods, respectively, while 2H-1-Benzopyran-2-one, Bergapten, and Genistein were found in over 140 foods each. Lastly, Methylparaben (141 foods), Benzyl alcohol (139 foods), and camphor (124 foods) completed the top 20 list. These compounds, due to their frequent association with CYP2A6-related foods, may play significant roles in CYP2A6 modulation and represent promising candidates for further investigation.

### 3.3. Experimental Validation of Our Findings

Fourteen (14) of the aforementioned top 20 compounds were experimentally screened in vitro for 2A5 inhibition at 1 mM concentrations to validate the results and identify the most potent inhibitors. As shown in Table 2, four of the 14 compounds, namely, 1,8-cineole, benzaldehyde, camphor and geraniol, proved to be potent 2A5 inhibitors, achieving inhibition of >70%.

These 4 most potent compounds were then investigated and validated further by testing their inhibitory effect at different concentrations. As shown in Figure 2A, all 4 inhibited 2A5 in a concentration-dependent manner, with IC_50_ of 0.018, (95% CI 0.0153–0.0214), 0.011 (95% CI 0.00977–0.0123), 0.13 (95% CI 0.106–0.173), and 0.026 (95% CI 0.0213–0.0322) mM for 1,8-cineole, camphor, benzaldehyde and geraniol, respectively. At the lower concentration of 0.005 mM tested, the % inhibition of 2A5 was 25.00%, 33.75%, 24.66% and 5.34% for 1,8-cineole, camphor, geraniol and benzaldehyde, respectively (Figure 2B). Interestingly, when the 4 compounds were combined at a concentration of 0.005 mM each, the % inhibition achieved was 49.62%. In comparison, 8-methoxy psoralen, one of the most potent 2A6 inhibitors known, at a concentration of 0.005 mM achieved an inhibition of 73.44%.

### 3.4. Investigation of Foods Containing Experimentally Validated Compounds

Experimental validation confirmed four compounds with CYP2A5 inhibitory potential: geraniol, benzaldehyde, 1,8-cineole, and camphor. We further investigated CYP2A6-related foods containing these compounds using the FooDisNET database. Specifically, we identified 183 foods containing benzaldehyde, 152 for 1,8-cineole, 124 for camphor, and 195 for geraniol. A commonality analysis was then performed to identify foods that contain all four CYP2A6-inhibiting compounds. Figure 3 shows the 14 foods found to be common: Chinese cinnamon, Mandarin orange (Clementine, Tangerine), Turmeric, Fennel, Corn, Ginger, *Artemisia montana* (mountain mugwort), *Artemisia capillaris* (capillary wormwood), *Akebia trifoliata* (three-leaf chocolate vine), *Mosla chinensis* (Chinese mosquito plant), *Akebia quinata* (five-leaf chocolate vine), *Elsholtzia ciliata* (Vietnamese balm), *Artemisia princeps* (Japanese mugwort or yomogi) and *Artemisia argyi* (silvery wormwood or Chinese mugwort).

### 3.5. Dietary Occurrence and Human Exposure of the Validated Compounds

To better understand the dietary relevance of the validated compounds, we examined the published literature describing their occurrence in the food sources identified through FooDisNET. The reported concentrations of these compounds varied across the different food sources. For example, gas chromatography–mass spectrometry (GC–MS) analysis of ginger (*Zingiber officinale*) essential oil showed that 1,8-cineole and camphor accounted for 3.0 ± 0.1% and 0.2 ± 0.0% of the essential-oil composition, respectively. In turmeric (*Curcuma longa*) essential oil, 1,8-cineole accounted for 1.0 ± 0.0% [13]. Mandarin (*Citrus reticulata*), one of the 14 foods containing all four validated compounds, was also examined. Studies of mandarin peel essential oil reported both 1,8-cineole and geraniol, with concentrations reaching approximately 26% and 3.5%, respectively, depending on the cultivar and analytical conditions [14,15]. However, these values refer to the composition of the concentrated essential oil and should not be interpreted as concentrations in the whole fruit as consumed.

Additional quantitative data from ten *C. longa* rhizome samples, for which both essential-oil yield and corresponding GC–MS composition were reported, allowed estimation of the 1,8-cineole content in the rhizome. For each sample, the reported essential-oil yield was first used to estimate the amount of essential oil obtained from 100 g of rhizome. The corresponding percentage of 1,8-cineole determined by GC–MS was then applied to this amount to estimate the quantity of 1,8-cineole present in 100 g of rhizome. Across the ten samples, the estimated 1,8-cineole content ranged from approximately 11.1 to 42.5 mg per 100 g of rhizome, corresponding to approximately 0.111–0.425 mg/g of turmeric [16].

To place this estimate in a dietary context, we used consumption data from the Korean National Health and Nutrition Examination Survey (2008–2012). In this Korean population aged 10–69 years, curcumin-containing foods were consumed mainly as curry powder and ready-made curry products, and the mean turmeric intake among consumers was estimated at 0.47 g/day. Combining this reported daily turmeric intake with the estimated 1,8-cineole content of 0.111–0.425 mg/g resulted in a theoretical daily intake of approximately 0.052–0.200 mg of 1,8-cineole from turmeric [17].

These published analytical data also provide independent support for several of the food–compound associations identified through FooDisNET. However, the estimated dietary exposure should be interpreted cautiously, as the reported turmeric intake was derived primarily from the consumption of curry products, whereas the 1,8-cineole content was estimated from analyses of turmeric rhizomes. Furthermore, this estimate does not account for food processing, variability in compound composition, or bioavailability.

To provide further context for the concentrations tested in vitro, we next considered the available human pharmacokinetic data. Consumption of 1.02 mg of 1,8-cineole (19 μg/kg body weight) resulted in a maximum plasma concentration of 19 nmol/L (0.019 μM) for the parent compound, while the 2-hydroxy- and 9-hydroxy-1,8-cineole metabolites reached maximum plasma concentrations of 86 and 33 nmol/L, respectively [18]. These data provide information on systemic exposure following a relatively low oral intake. We therefore also examined human pharmacokinetic data following administration of a concentrated oral formulation.

In a human pharmacokinetic study, a single 300 mg dose of Myrtol standardized was administered to healthy volunteers under fasting and fed conditions. The formulation contained 46.35% eucalyptol (1,8-cineole), meaning that the administered 300 mg dose corresponded to approximately 139 mg of 1,8-cineole. The reported maximum plasma concentrations of 1,8-cineole were 167.60 ng/mL under fasting conditions and 518.89 ng/mL after a meal. To express these values in the same units as those used in the present study, the reported plasma concentrations were converted to micromolar concentrations using the molecular weight of 1,8-cineole (154.25 g/mol), resulting in approximately 1.09 μM and 3.36 μM under fasting and fed conditions, respectively [19]. These findings demonstrate that this concentrated oral formulation produced measurable micromolar systemic exposure to 1,8-cineole in humans. The oral use of concentrated 1,8-cineole formulations in human clinical studies has also been described [20].

Human oral-exposure data have also been reported for geraniol [21]. In a human oral-exposure study, 25 or 250 mg of geraniol was administered orally to human volunteers, and urinary metabolites were monitored for 72 h. Maximum urinary concentrations of the measured metabolites were observed within 1–5 h after administration, with estimated elimination half-lives of approximately 2–4 h. Based on urinary biomonitoring data from occupationally non-exposed adults, the same study estimated a mean daily geraniol uptake of 1.43 mg [21]. However, plasma concentrations of the parent compound were not reported, and therefore the circulating geraniol concentrations resulting from these oral doses could not be determined.

We also examined human studies involving concentrated oral formulations of some of the foods and herbs identified in our analysis. One human crossover study evaluated five turmeric formulations in 30 healthy adults, using single oral doses ranging from 300 to 1515 mg, and demonstrated that the type of formulation markedly influenced systemic exposure to curcuminoids [22]. Similarly, standardized ginger extract has been administered to 27 healthy volunteers at single oral doses ranging from 100 mg to 2 g [23]. Although these studies evaluated curcuminoids and gingerols/shogaols rather than the specific compounds validated in the present study, they demonstrate that turmeric and ginger have been administered as concentrated oral formulations in human studies.

For camphor and benzaldehyde, suitable quantitative human plasma exposure data following dietary intake or oral supplementation that would allow direct assessment of systemic exposure were not identified. Therefore, reliable estimates of the dietary or supplemental amounts required to achieve specific circulating concentrations could not be derived for these compounds.

### 3.6. Potential Dietary Combinations of Foods Containing CYP2A6-Inhibitory Compounds

The fourteen foods identified as containing all four experimentally validated CYP2A6-inhibitory compounds span a broad range of culinary traditions. Several of these (Chinese cinnamon, mandarin orange/clementine/tangerine, turmeric, fennel, corn, and ginger) are ingredients of well-characterized global dietary patterns, most notably the Mediterranean Diet and certain Asian dietary traditions, including the Traditional Chinese Diet.

In light of the dietary occurrence and human exposure data described above, the co-occurrence of these compounds within specific foods and dietary patterns is of interest for exploring how dietary sources, potentially complemented by concentrated formulations, could contribute to overall exposure. The dietary combinations discussed below should nevertheless be considered as potential strategies for further investigation rather than as established nutritional recommendations for CYP2A6 inhibition.

### 3.7. The Mediterranean Diet

Among the fourteen foods identified in this study, several are well-established elements of the Mediterranean culinary tradition: mandarin oranges and clementines, fennel, corn, ginger, and cinnamon, with turmeric increasingly incorporated into contemporary Mediterranean cooking. The Mediterranean Diet (MD) is characterized by high intake of vegetables, fruits, legumes, whole grains, nuts, fish, and extra-virgin olive oil, with moderate dairy and wine consumption and low red meat intake [24]. It is rich in dietary fiber, monounsaturated fatty acids, omega-3 fatty acids, and a broad spectrum of antioxidants including polyphenols, carotenoids, tocopherols, and vitamin C, all of which are relevant in the context of smoking cessation since smokers typically present with depleted antioxidant status, elevated systemic inflammation, and impaired endothelial function [25,26].

### 3.8. The Traditional Chinese Diet

The Traditional Chinese Diet (TCD) is the dietary pattern most directly aligned with the identified inhibitory foods, given the centrality of Chinese cinnamon, ginger, turmeric, fennel, and corn in Chinese culinary traditions, and the longstanding use of *Artemisia* species (*A. capillaris*, *A. princeps*, *A. argyi*), *Elsholtzia ciliata*, and *Akebia* species in Traditional Chinese Medicine [27,28,29]. Characterized by high intake of rice, leafy vegetables, wheat, legumes, soy products, corn, and aromatic spices, with moderate fish and poultry consumption (and other foods depending on regional variation), the TCD is a health-protecting dietary pattern [30]. Habitual consumption of the identified spices may therefore contribute to dietary exposure to geraniol, benzaldehyde, 1,8-cineole, and camphor. Since more urbanized, Westernized variants of the Chinese diet often feature higher sodium, refined carbohydrates, and less favorable fatty acid profiles [31]; recommendations should emphasize the traditional, plant-based pattern.

A key feature of both dietary patterns is the habitual co-consumption of multiple inhibitory foods within the same meal, an aspect directly relevant to the additive effect observed in the current study, where combining all four validated compounds at 0.005 mM each achieved 49.62% inhibition of CYP2A5—substantially greater than individual compounds alone. This additive effect highlights the potential importance of evaluating combinations of compounds rather than isolated compounds. While the concentrations achievable through normal dietary intake remain uncertain, the human exposure data described above indicate that concentrated oral formulations can increase systemic exposure to at least some of these compounds. This supports further investigation of combined dietary and formulation-based approaches. Of note, these two dietary frameworks not only offer the broadest coverage of the fourteen identified foods, but also provide established nutritional benefits, including being rich in antioxidants, dietary fiber, and anti-inflammatory phytochemicals, all of which are relevant to overall health and smoking cessation.

### 3.9. Strategies to Maximize Benefit

Several practical dietary strategies may help individuals adopting either a Mediterranean or Traditional Chinese dietary pattern to maximize the potential CYP2A6-inhibitory and broader health benefits during smoking cessation.

Firstly, given the additive CYP2A5 inhibition observed when the four compounds were combined in vitro, incorporating multiple dietary sources of geraniol, benzaldehyde, 1,8-cineole, and camphor within the same meal could be considered as a potential strategy to increase their combined dietary exposure. Practically, this could be achieved through Mediterranean-inspired meals pairing citrus fruit such as mandarin or clementine as a dessert or in dressings, ginger-spiced grains, fennel in salads, and cinnamon-flavored herbal teas. A similar approach can be applied within a Chinese dietary framework, combining ginger and cinnamon in broths, fennel in spice blends, and corn as a staple grain.

Second, culinary preparation methods matter. The volatile monoterpene compounds identified in this study (1,8-cineole, geraniol, camphor) are sensitive to heat and may be partially degraded by prolonged high-temperature cooking. To preserve bioactive compound content, gentler cooking methods are recommended: using fresh ginger rather than dried or pre-cooked forms, adding cinnamon to hot beverages rather than baked goods subjected to prolonged oven temperatures, and incorporating fresh fennel leaves into salads or as garnish rather than cooking them for extended periods. Geraniol content is better preserved in foods consumed raw or lightly processed [32].

Third, consistency and dietary frequency may also be important considerations alongside the total level of exposure. Given that CYP2A6 enzyme activity is a dynamic and continuously regulated process, maintaining a steady dietary intake of inhibitory-compound-rich foods across multiple daily eating occasions could contribute to more consistent dietary exposure. This aligns with the principles of both the MD and TCD, which favor frequent consumption of diverse plant foods across all meals rather than reliance on supplements or isolated extracts [24]. Such dietary incorporation could potentially be complemented by appropriately characterized concentrated oral formulations or supplements to further increase overall exposure to the identified compounds.

Fourth, it is important to acknowledge that interindividual variability in CYP2A6 genetic polymorphisms may influence the extent to which dietary inhibitors modulate nicotine metabolism. Normal metabolisers (NMs) carry two functional CYP2A6 alleles and account for approximately 80–90% of European populations, while reduced-function and loss-of-function alleles are more prevalent in East Asian populations [33]. Dietary CYP2A6 inhibition may be more pharmacologically meaningful in NMs who have a higher baseline enzymatic activity to inhibit, while slow metabolisers—who are more prevalent in Asian populations—may already have a naturally lower rate of nicotine clearance and may therefore derive less additional benefit from dietary inhibition strategies.

## 4. Discussion

Nicotine metabolism plays a central role in tobacco dependence, with CYP2A6 acting as the primary enzyme responsible for nicotine clearance [34]. Individuals with higher CYP2A6 activity metabolize nicotine more rapidly and tend to smoke more frequently to maintain nicotine concentrations, reinforcing addiction. Consequently, strategies aimed at modulating CYP2A6 activity have attracted growing interest as complementary approaches to smoking cessation [4]. In this study, we combined a computational food-compound prioritization strategy with experimental validation to identify naturally occurring, diet-derived compounds with potential relevance to CYP2A6-mediated nicotine metabolism.

Using the FooDisNET database, we systematically identified foods associated with CYP2A6 and analyzed their constituent compounds, enabling data-driven prioritization of candidate compounds for experimental evaluation. The majority voting approach highlighted compounds frequently occurring across multiple CYP2A6-related foods, thereby providing a systematic basis for prioritizing bioactive compounds for further experimental investigation.

Experimental validation using a liver microsome-based assay confirmed that four compounds—1,8-cineole, camphor, benzaldehyde, and geraniol—exhibited strong inhibitory activity against CYP2A5, the murine orthologue of human CYP2A6. All four compounds demonstrated concentration-dependent inhibition with IC_50_ values in the micromolar range, supporting the robustness of the computational prioritization strategy. Although CYP2A5 is not identical to CYP2A6, it is widely accepted as a functional orthologue and is commonly used for preclinical assessment of CYP2A6-related metabolic processes. Both enzymes are involved in nicotine metabolism and share close structural similarity, with approximately 84% amino acid sequence similarity [35,36,37,38]. Direct kinetic comparisons have demonstrated that both CYP2A5 and CYP2A6 efficiently catalyze nicotine 5′-oxidation, although with different kinetic characteristics [36]. Furthermore, in vivo evidence from a Cyp2a5-null mouse model demonstrated markedly reduced hepatic nicotine 5′-hydroxylation activity and slower systemic clearance of nicotine and cotinine compared with wild-type mice, supporting a major role for CYP2A5 in murine nicotine metabolism [39]. Together with previous studies demonstrating CYP2A5-mediated nicotine metabolism in vivo [37,38], these findings support the biological relevance of CYP2A5 as a preclinical model for investigating CYP2A6-related nicotine metabolism. Nevertheless, differences between the two enzymes may influence inhibitor potency and IC_50_ values; therefore, the inhibitory effects identified here require confirmation using human liver microsomes or recombinant human CYP2A6 systems.

Notably, when tested at low concentrations, individual compounds produced modest inhibition, whereas their combination resulted in a substantially greater inhibitory effect. This observation suggests an additive effect under the experimental conditions tested and highlights the potential importance of interactions among multiple bioactive compounds. However, whether such combined effects occur at concentrations achievable in humans through dietary intake and/or concentrated oral formulations remains to be established.

The additional dietary and human-exposure assessment provides important context for interpreting the translational relevance of these findings. For 1,8-cineole, the theoretical dietary intake estimated from turmeric (approximately 0.052–0.200 mg/day) was derived by combining published quantitative compositional data for *C. longa* rhizomes with a reported mean turmeric intake of 0.47 g/day among consumers of curcumin-containing foods [16,17]. Following an oral dose of 1.02 mg of 1,8-cineole in a human pharmacokinetic study, the maximum plasma concentration of the parent compound was 0.019 μM [18]. Importantly, higher systemic exposure has been reported following administration of a concentrated oral formulation. A 300 mg dose of Myrtol standardized, corresponding to approximately 139 mg of 1,8-cineole, resulted in maximum plasma concentrations of approximately 1.09 μM under fasting conditions and 3.36 μM after a meal [19]. These findings indicate that systemic exposure to 1,8-cineole depends substantially on the amount and formulation administered and suggest that concentrated oral formulations may provide a means of increasing exposure beyond that expected from normal dietary intake alone. For geraniol, camphor, and benzaldehyde, the limited availability of quantitative human plasma exposure data prevented equivalent comparisons. Collectively, these findings suggest that dietary sources may be complemented by concentrated oral formulations to increase overall exposure to the identified compounds. However, further human pharmacokinetic studies are needed to determine the circulating concentrations achievable for each compound and to establish whether these concentrations are sufficient to modulate human CYP2A6 activity.

Several of the compounds identified and experimentally validated in this study have previously been implicated in the modulation of cytochrome P450–mediated metabolism, providing additional biological context for their experimentally observed inhibitory effects. Benzaldehyde belongs to the class of aromatic aldehydes, which includes cinnamon-derived compounds such as trans-cinnamaldehyde and 2-methoxycinnamaldehyde that have been shown to inhibit CYP2A6 activity in a time-dependent manner [8]. Monoterpenes such as 1,8-cineole and camphor have been reported to interact with drug-metabolizing enzymes and modulate cytochrome P450 activity involved in xenobiotic clearance [40]. Moreover, geraniol, a monoterpene alcohol widely present in edible plants, is a key precursor of structurally diverse bioactive derivatives whose biosynthesis is largely mediated by cytochrome P450 enzymes, highlighting its close association with CYP-dependent oxidative metabolism [41].

An additional consideration is the CYP selectivity of the identified compounds. Natural bioactive compounds may interact with multiple CYP isoforms, potentially affecting xenobiotic metabolism and raising the possibility of food–drug interactions. This is particularly relevant for 1,8-cineole, which has been shown to undergo CYP3A4-mediated metabolism in human liver microsomes and recombinant human CYP systems [42], indicating that its interactions with CYP enzymes are not restricted to CYP2A-related pathways. Camphor has also been reported to undergo CYP2A6-mediated oxidation in human liver microsomes [43]. However, the four compounds were not systematically evaluated against a panel of major human CYP isoforms in the present study. Therefore, their selectivity toward CYP2A6-related metabolism cannot be established, and potential interactions with other CYP-mediated pathways cannot be excluded. Comparative assessment against major human CYP isoforms, including CYP1A2, CYP2B6, CYP2C9, CYP2D6, and CYP3A4, would be required to characterize their selectivity and potential food–drug interaction risk.

Mapping the validated inhibitory compounds back to their food sources revealed several commonly consumed foods and traditional medicinal plants that contain multiple compounds with inhibitory activity against CYP2A5 and potential relevance to CYP2A6-mediated metabolism. Foods such as cinnamon, citrus fruits, ginger, and fennel are enriched in monoterpenes and aromatic compounds, including geraniol, benzaldehyde, 1,8-cineole, and camphor, many of which have documented interactions with cytochrome P450 enzymes [44,45]. For example, published compositional analyses of mandarin (*Citrus reticulata*), one of the 14 foods containing all four validated compounds, reported the presence of both 1,8-cineole and geraniol in mandarin peel essential oil [14,15]. In addition, compounds derived from *Artemisia* species have been widely reported to interact with cytochrome P450–mediated metabolic pathways. *Artemisia capillaris* Thunb., traditionally used for the treatment of jaundice, has been shown to regulate the expression of murine cytochrome P450 2a5 (Cyp2a5), the functional orthologue of human CYP2A6, through activation of the constitutive androstane receptor (CAR) [46]. In contrast, *Artemisia annua* L., best known for its antimalarial constituent artemisinin, has been reported to modulate the activity and expression of major drug-metabolizing enzymes, including CYP2B6 and CYP3A4 [47].

From a dietary patterns perspective, both the Mediterranean Diet and the Traditional Chinese Diet provide relevant frameworks for contextualizing the identified foods within broader dietary habits. Both patterns share established nutritional properties—high antioxidant content, dietary fiber, and diverse anti-inflammatory phytochemicals—that independently benefit smokers, who typically present with depleted antioxidant status, elevated systemic inflammation, and impaired endothelial function [25,26]. Tobacco use depletes plasma vitamin C and β-carotene, and smokers require substantially higher vitamin C intakes than non-smokers—approximately two-fold greater—to reach equivalent serum concentrations [48]. Both dietary patterns, through their emphasis on plant foods, are well positioned to replenish these deficits. Although several of the identified foods can be incorporated within these dietary patterns, the present findings do not establish that their consumption, either individually or in combination, can achieve systemic concentrations sufficient to inhibit CYP2A6 in humans. However, the human exposure data discussed above indicate that concentrated oral formulations may provide an additional means of increasing systemic exposure to at least some of the identified compounds. Therefore, these dietary patterns, potentially complemented by appropriately characterized concentrated formulations, provide a framework for further investigating whether combined dietary and supplemental exposure to the identified compounds could contribute to the modulation of nicotine metabolism.

Beyond dietary modulation, pharmacological smoking cessation aids such as varenicline and cytisine act primarily as partial agonists of the α4β2 nicotinic acetylcholine receptor rather than through inhibition of nicotine-metabolizing enzymes. Notably, both agents undergo minimal hepatic metabolism: varenicline is excreted largely unchanged in urine (~92%) and does not inhibit or induce CYP450 enzymes, including CYP2A6 [49], while cytisine is predominantly unchanged via renal excretion, with no significant hepatic biotransformation reported in human pharmacokinetic studies [50]. This distinguishes them pharmacokinetically from the diet-derived compounds investigated in the present study for their potential relevance to CYP2A6-mediated nicotine metabolism and suggests that CYP2A6-mediated pharmacokinetic interactions with these smoking-cessation agents need further investigation. Nevertheless, CYP2A6 activity—commonly assessed via the nicotine metabolite ratio (NMR)—has been shown to predict differential treatment response: individuals with slower CYP2A6-mediated nicotine metabolism showed no significant efficacy advantage of varenicline over nicotine patch, whereas normal metabolizers showed greater smoking-cessation efficacy with varenicline than with nicotine patch [51]. These findings indicate that nicotine metabolic phenotype may be relevant to treatment response during smoking cessation. Therefore, dietary or supplementation-based approaches involving compounds with the potential to modulate CYP2A6 activity could theoretically alter nicotine clearance while nicotine exposure persists, although whether such modulation is sufficient to influence treatment response remains unknown. Further investigation would be required to establish whether dietary or supplementation-based approaches targeting CYP2A6-mediated nicotine metabolism interact with smoking-cessation or smoking reduction pharmacotherapy.

Several limitations of the current study should be acknowledged. First, the initial identification of CYP2A6-related foods and compounds was based on curated database information, and as with any database-driven approach, the results depend on the completeness and accuracy of the underlying annotations. Consequently, some relevant food–compound associations may be underrepresented or missing. Second, experimental validation was performed using murine liver microsomes. Although CYP2A5 is a well-established functional orthologue of human CYP2A6, differences between the two enzymes may influence inhibitor potency and IC_50_ values. Therefore, the inhibitory effects identified in the present study should be considered preclinical and require confirmation using human liver microsomes or recombinant human CYP2A6 systems. Furthermore, the selectivity of the four validated compounds toward CYP2A6-related metabolism was not assessed against other major human CYP isoforms. Therefore, potential effects on other CYP-mediated pathways and the associated potential risk of food–drug interactions cannot be excluded.

An additional limitation of the present study is that the in vitro microsome assay measured endpoint inhibition of coumarin 7-hydroxylation following a 30 min incubation, without incorporating an NADPH pre-incubation step. Consequently, the current experimental design does not differentiate between reversible inhibition and mechanism-based/time-dependent inhibition. Time-dependent inhibition is well documented for several CYP2A6 modulators, including dietary aromatic aldehydes such as cinnamaldehyde derivatives [8] and natural monoterpenoids such as menthofuran [52] and is crucial for predicting long-term in vivo inactivation and kinetic interactions. Of note, 1,8-cineole, camphor, and geraniol are monoterpenoids, while benzaldehyde is an aromatic aldehyde, making these compounds good candidates for mechanism-based inactivation of the 2A5/2A6 enzymes, which, however, remains to be determined in future studies in recombinant human CYP2A6.

Third, the concentrations tested may exceed systemic concentrations achievable following normal dietary intake, as illustrated by the available human exposure data for 1,8-cineole. However, human pharmacokinetic data also demonstrate that concentrated oral formulations can increase systemic exposure to 1,8-cineole. Thus, concentrated formulations, potentially used alongside dietary sources, may provide an additional means of increasing exposure, although the concentrations achievable for each of the identified compounds remain to be established. Although an additive inhibitory effect was observed when the four compounds were combined in vitro, whether combined dietary and supplemental exposure can produce biologically meaningful CYP2A6 inhibition in vivo remains unknown. Moreover, quantitative human plasma exposure data were limited or unavailable for several of the validated compounds, preventing direct comparison between human systemic exposure and the experimentally effective concentrations. Further human pharmacokinetic studies are therefore needed to establish achievable systemic exposures and their relevance to CYP2A6 modulation. Finally, interindividual variability in CYP2A6 genetic polymorphisms may influence responsiveness to dietary and supplementation-based strategies and should be considered in future personalized nutrition and precision-health studies.

Several strengths of the present study should also be highlighted. To our knowledge, this is among the first studies to combine a food-centered computational prioritization framework with biochemical validation to identify diet-derived modulators of nicotine metabolism. The integration of database-driven compound prioritization, concentration-dependent experimental validation, food-source mapping, and literature-based dietary and human exposure assessment provides a multi-level framework for evaluating both the biological activity and the potential translational relevance of the identified compounds. Importantly, the inclusion of human exposure data provides additional context for considering how dietary sources, potentially complemented by concentrated oral formulations, could contribute to increasing exposure to the identified compounds.

## 5. Conclusions

In conclusion, this study demonstrates that integrating computational food–compound analysis with experimental validation provides an effective framework for identifying natural compounds with inhibitory activity toward CYP2A5, the functional orthologue of human CYP2A6. The identified compounds and their corresponding food sources represent promising candidates for further investigation as supportive, nutrition-based approaches to modulating nicotine metabolism and aiding smoking cessation. Importantly, available human pharmacokinetic evidence indicates that concentrated oral formulations can increase systemic exposure to at least some of the identified compounds, highlighting the potential of combining dietary sources with appropriately characterized supplements or concentrated formulations to enhance overall exposure. This integrated dietary and supplementation-based strategy therefore represents a promising avenue for future investigation. Further validation in human CYP2A6 systems and human pharmacokinetic studies will be important to establish achievable circulating concentrations and determine their potential to modulate nicotine metabolism in humans.

## Figures and Tables

**Figure 1:**
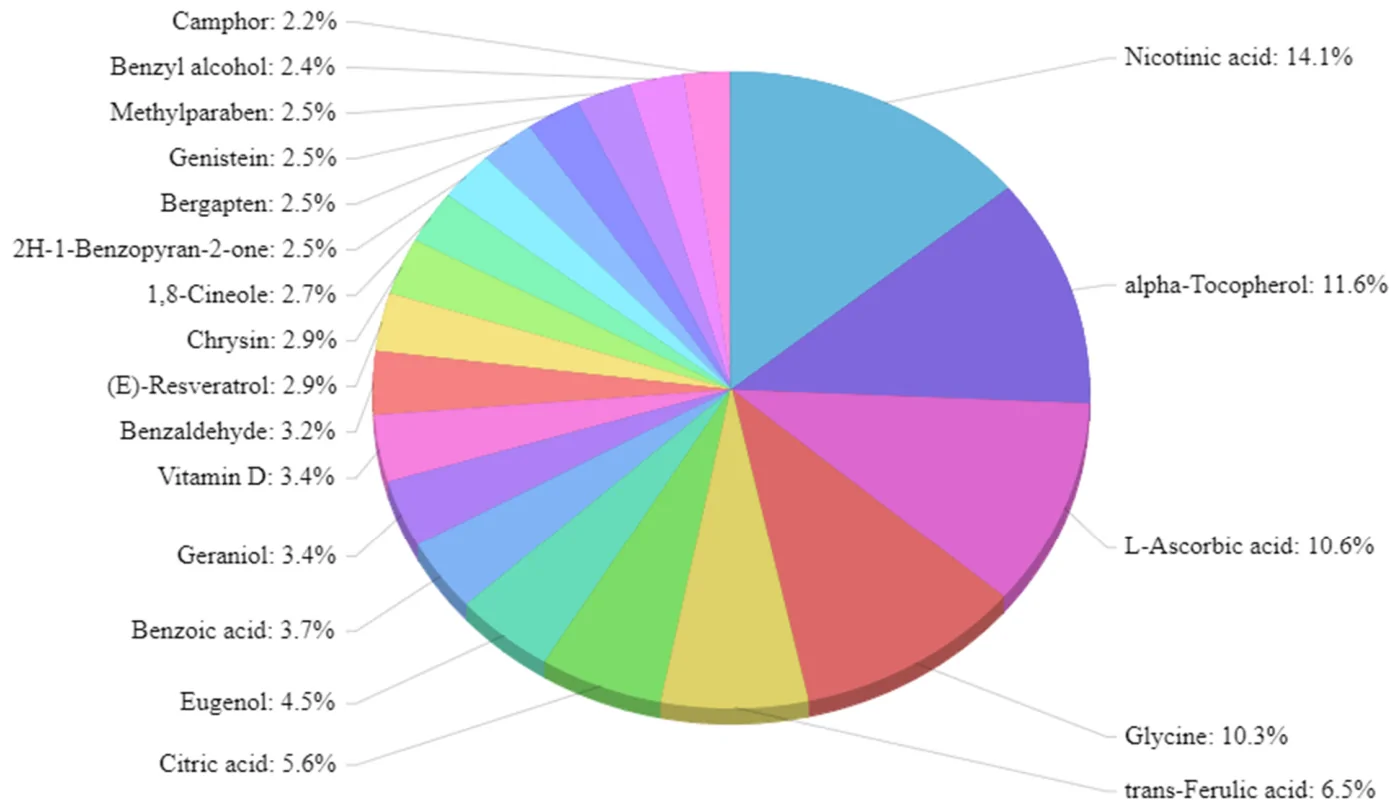
Pie chart illustrating the results of the majority voting analysis for compounds associated with CYP2A6-related foods. The chart displays the top 20 compounds based on their frequency across CYP2A6-related foods. The size of each segment represents the proportion of foods associated with each compound.

**Figure 2:**
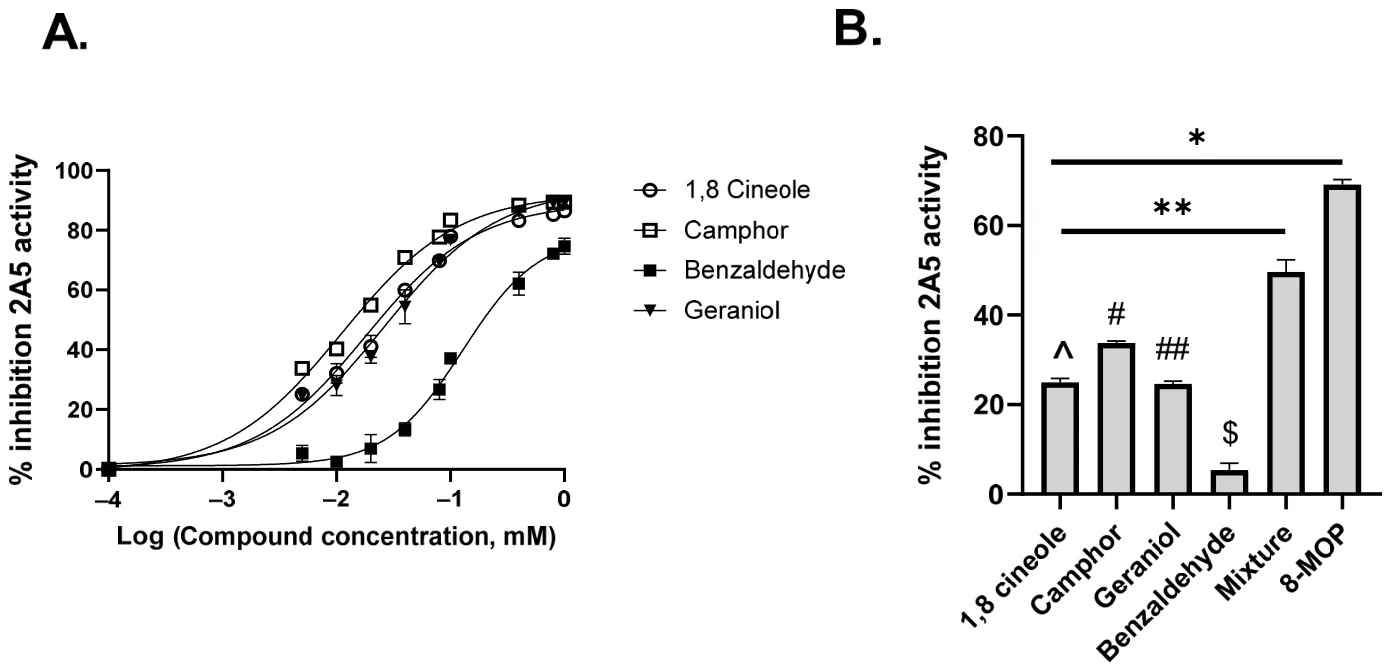
Inhibition of CYP2A5 enzyme by test compounds. (**A**). Concentration-dependent (0.005–1 mM) inhibition of CYP2A5 by the 4 tested compounds. A Non-linear regression of log (compound concentration/inhibitor)—vs. response—Variable slope (four parameters) fit was performed from *n* = 3. (**B**). Inhibition of CYP2A5 enzyme by the tested compounds at 0.005 mM concentration alone or mixed. 8-methoxypsoralen (8-MOP) was shown as a positive control. Data are presented as means ± SEM (*n* = 3). *, ** *p* < 0.05 vs. all treatments, ^ *p* < 0.05 vs. camphor and benzaldehyde, $ *p* < 0.05 vs. camphor, geraniol and 1,8-cineole, # *p* < 0.05 vs. geraniol, 1,8-cineole and benzaldehyde, ## *p* < 0.05 vs. 1,8-cineole, benzaldehyde and camphor.

**Figure 3:**
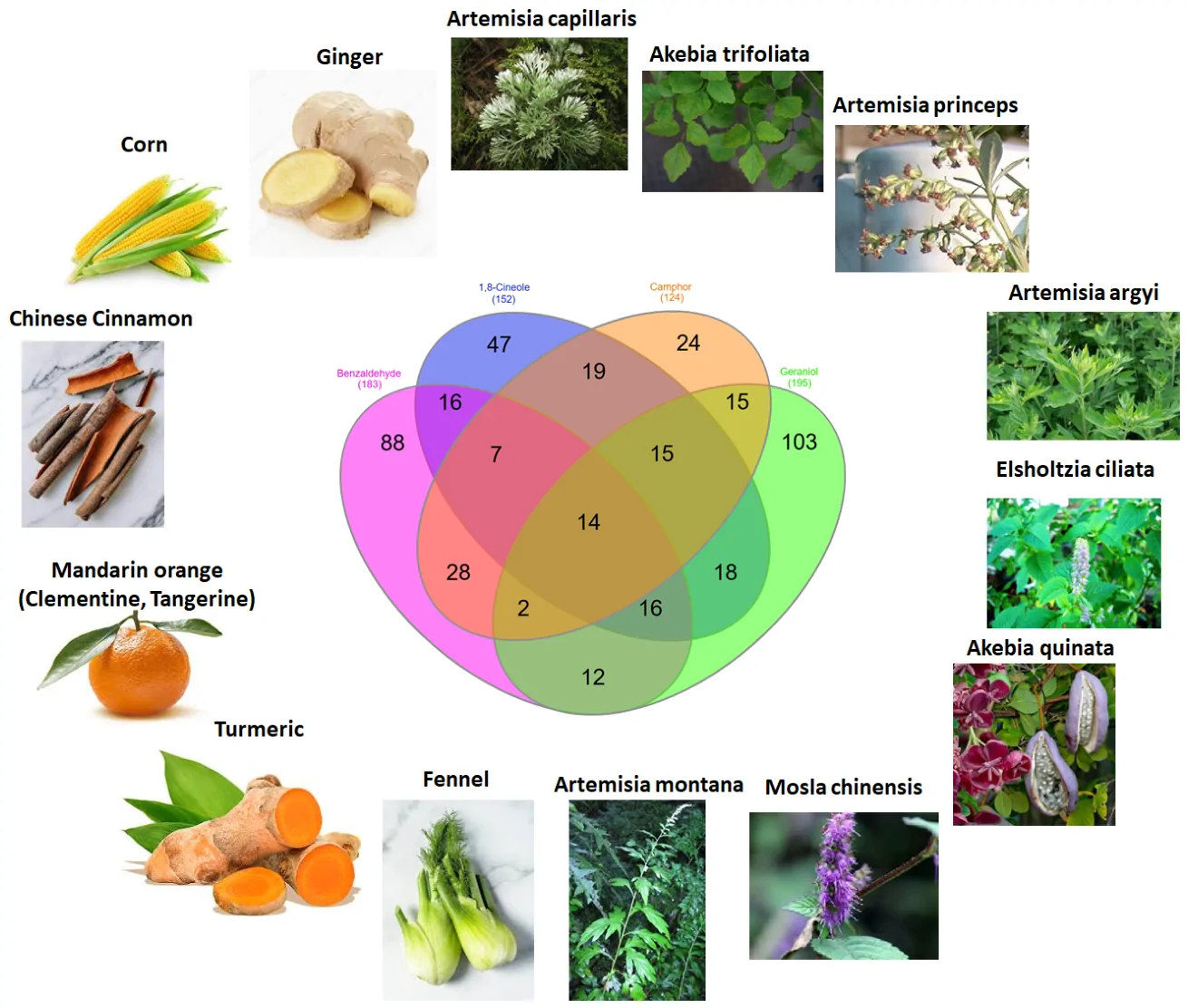
Venn diagram showing the comparison of CYP2A6-related foods containing the four experimentally validated CYP2A5-inhibiting compounds: benzaldehyde, 1,8-cineole, camphor, and geraniol. Numbers within each region indicate the number of foods unique to each compound or shared among the corresponding compounds. The food images shown around the Venn diagram represent the 14 foods containing all four compounds.

**Table 1:** Top 20 CYP2A6-related foods based on the number of associated compounds.

BXGF ID	Food Name	Scientific Name	Food Group	Number of Compounds Identified in Cyp2a6-Associated Foods *
**BXGF00205**	Corn	*Zea mays*	Cereals and cereal products	27
**BXGF00206**	Ginger	*Zingiber officinale*	Herbs and Spices	23
**BXGF00038**	Tea	*Camellia sinensis*	Teas	22
**BXGF00050**	Chinese cinnamon	*Cinnamomum aromaticum*	Herbs and Spices	22
**BXGF00082**	Fennel	*Foeniculum vulgare*	Herbs and Spices	21
**BXGF00057**	Sweet orange	*Citrus sinensis*	Fruits	20
**BXGF00072**	Wild carrot	*Daucus carota*	Vegetables	19
**BXGF00230**	Carrot	*Daucus carota* ssp. *sativus*	Vegetables	17
**BXGF00818**	Herbs and Spices		Herbs and Spices	17
**BXGF00204**	Common grape	*Vitis vinifera*	Fruits	17
**BXGF00056**	Mandarin orange (Clementine, Tangerine)	*Citrus reticulata*	Fruits	17
**BXGF04842**	Angelica acutiloba	*Angelica acutiloba*	Medicinal Plant	16
**BXGF00131**	Parsley	*Petroselinum crispum*	Herbs and Spices	16
**BXGF02049**	Angelica sinensis	*Angelica sinensis*	Medicinal Plant	16
**BXGF00041**	Papaya	*Carica papaya*	Fruits	16
**BXGF00182**	Cocoa bean	*Theobroma cacao*	Cocoa and cocoa products	16
**BXGF00823**	Fruits		Fruits	16
**BXGF00013**	Dill	*Anethum graveolens*	Herbs and Spices	16
**BXGF00173**	Garden tomato (var.)	*Solanum lycopersicum* var. *lycopersicum*	Vegetables	16
**BXGF00081**	Fig	*Ficus carica*	Fruits	16

* The number refers to compounds retrieved from FooDisNET for each CYP2A6-associated food and does not imply experimentally validated CYP2A6 inhibition.

**Table 2:** Initial screening of compounds for inhibition of CYP2A5 activity at 1 mM concentration. Data are presented as mean ± standard error of the mean (SEM), with *n* = 3 replicates.

	Test Compound	% Inhibition of CYP2A5 Activity
1	Geraniol	74.68 ± 2.6
2	1,8-cineole	75.44 ± 0.36
3	Eugenol	38.31 ± 0.21
4	Benzaldehyde	71.65 ± 0.64
5	Tocopherol	17.30 ± 14.4
6	Glycine	8.59 ± 1.66
7	Ascorbic acid	17.56 ± 1.05
8	Citric acid	19.84 ± 0.16
9	Camphor	75.74 ± 1.3
10	Benzoic acid	19.65 ± 3.38
11	Quercetin	29.58 ± 6.34
12	Ferulic acid	0
13	Resveratrol	25.69 ± 1.69
14	Nicotinic Acid	5.68 ± 2.06

Note: Highlighted values indicate compounds showing >70% inhibition of CYP2A5 activity.

## Data Availability

The data presented in this study are included in the article and its Supplementary Materials. Supplementary File S1 contains the 3078 CYP2A6-related foods identified through the FooDisNET database search, while Supplementary File S2 contains the 196 unique compounds identified across these CYP2A6-related foods.

## Supplementary Materials

The following supporting information can be downloaded at: https://www.mdpi.com/article/10.3390/nu18193162/s1. File S1: CYP2A6-related foods identified through the FooDisNET database search; File S2: Unique compounds identified across the CYP2A6-related foods.

## References

[1] St Claire S., Fayokun R., Commar A., Schotte K., Prasad V.M. (2020). The World Health Organization’s World No Tobacco Day 2020 Campaign Exposes Tobacco and Related Industry Tactics to Manipulate Children and Young People and Hook a New Generation of Users. J. Adolesc. Health.

[2] Benowitz N.L. (2009). Pharmacology of nicotine: Addiction, smoking-induced disease, and therapeutics. Annu. Rev. Pharmacol. Toxicol..

[3] Goyal N., Sridhar J., Do C., Bratton M., Shaik S., Jiang Q., Foroozesh M. (2021). Identification of CYP 2A6 inhibitors in an effort to mitigate the harmful effects of the phytochemical nicotine. J. Cancer Metastasis Treat..

[4] Tanner J.A., Tyndale R.F. (2017). Variation in CYP2A6 Activity and Personalized Medicine. J. Pers. Med..

[5] Tzoupis H., Papavasileiou K.D., Papatzelos S., Mavrogiorgis A., Zacharia L.C., Melagraki G., Afantitis A. (2024). Systematic Review of Naturally Derived Substances That Act as Inhibitors of the Nicotine Metabolizing Enzyme Cytochrome P450 2A6. Int. J. Mol. Sci..

[6] Ding K., Jiang W., Zhan W., Xiong C., Chen J., Wang Y., Jia H., Lei M. (2023). The therapeutic potential of quercetin for cigarette smoking-induced chronic obstructive pulmonary disease: A narrative review. Ther. Adv. Respir. Dis..

[7] Bojic M., Kondza M., Rimac H., Benkovic G., Males Z. (2019). The Effect of Flavonoid Aglycones on the CYP1A2, CYP2A6, CYP2C8 and CYP2D6 Enzymes Activity. Molecules.

[8] Espiritu M.J., Chen J., Yadav J., Larkin M., Pelletier R.D., Chan J.M., Gc J.B., Natesan S., Harrelson J.P. (2020). Mechanisms of Herb-Drug Interactions Involving Cinnamon and CYP2A6: Focus on Time-Dependent Inhibition by Cinnamaldehyde and 2-Methoxycinnamaldehyde. Drug Metab. Dispos..

[9] Prasopthum A., Pouyfung P., Sarapusit S., Srisook E., Rongnoparut P. (2015). Inhibition effects of Vernonia cinerea active compounds against cytochrome P450 2A6 and human monoamine oxidases, possible targets for reduction of tobacco dependence. Drug Metab. Pharmacokinet..

[10] Guttman Y., Kerem Z. (2022). Dietary Inhibitors of CYP3A4 Are Revealed Using Virtual Screening by Using a New Deep-Learning Classifier. J. Agric. Food Chem..

[11] Lin C.-Y., Lee J.-Y., Huang S.-H., Hsu Y.-C., Hsu N.-Y., Yang J.-M. FooDisNET: A database of food-compound-protein-disease associations. Proceedings of the 2020 IEEE 20th International Conference on Bioinformatics and Bioengineering (BIBE).

[12] Abu-Bakar A., Hakkola J., Juvonen R., Rahnasto-Rilla M., Raunio H., Lang M.A. (2013). Function and regulation of the Cyp2a5/CYP2A6 genes in response to toxic insults in the liver. Curr. Drug Metab..

[13] Ibanez M.D., Blazquez M.A. (2019). Ginger and Turmeric Essential Oils for Weed Control and Food Crop Protection. Plants.

[14] Chutia M., Bhuyan P.D., Pathak M.G., Sarma T.C., Boruah P. (2009). Antifungal activity and chemical composition of Citrus reticulata Blanco essential oil against phytopathogens from North East India. LWT-Food Sci. Technol..

[15] Bourgou S., Rahali F.Z., Ourghemmi I., Saidani Tounsi M. (2012). Changes of peel essential oil composition of four Tunisian citrus during fruit maturation. Sci. World J..

[16] Setzer W.N., Duong L., Poudel A., Mentreddy S.R. (2021). Variation in the Chemical Composition of Five Varieties of Curcuma longa Rhizome Essential Oils Cultivated in North Alabama. Foods.

[17] Kwon Y. (2014). Estimation of curcumin intake in Korea based on the Korea National Health and Nutrition Examination Survey (2008–2012). Nutr. Res. Pract..

[18] Horst K., Rychlik M. (2010). Quantification of 1,8-cineole and of its metabolites in humans using stable isotope dilution assays. Mol. Nutr. Food Res..

[19] Zhao T.T., Zhu L.L., Chen M., Zhou Q. (2016). Is it appropriate regarding patient preference to take Myrtol standardized enteric-coated soft capsules after a meal rather than at fasted state? A food-drug pharmacokinetic interaction study in healthy Chinese volunteers. Patient Prefer. Adherence.

[20] Hoch C.C., Petry J., Griesbaum L., Weiser T., Werner K., Ploch M., Verschoor A., Multhoff G., Bashiri Dezfouli A., Wollenberg B. (2023). 1,8-cineole (eucalyptol): A versatile phytochemical with therapeutic applications across multiple diseases. Biomed. Pharmacother..

[21] Jager T., Koch H.M., Leibold E., Bader M. (2023). Human Metabolism and Urinary Elimination Kinetics of the Fragrance Geraniol after Oral Dosage. Chem. Res. Toxicol..

[22] Fanca-Berthon P., Tenon M., Bouter-Banon S.L., Manfre A., Maudet C., Dion A., Chevallier H., Laval J., van Breemen R.B. (2021). Pharmacokinetics of a Single Dose of Turmeric Curcuminoids Depends on Formulation: Results of a Human Crossover Study. J. Nutr..

[23] Zick S.M., Djuric Z., Ruffin M.T., Litzinger A.J., Normolle D.P., Alrawi S., Feng M.R., Brenner D.E. (2008). Pharmacokinetics of 6-gingerol, 8-gingerol, 10-gingerol, and 6-shogaol and conjugate metabolites in healthy human subjects. Cancer Epidemiol. Biomark. Prev..

[24] Estruch R., Ros E., Salas-Salvado J., Covas M.I., Corella D., Aros F., Gomez-Gracia E., Ruiz-Gutierrez V., Fiol M., Lapetra J. (2018). Primary Prevention of Cardiovascular Disease with a Mediterranean Diet Supplemented with Extra-Virgin Olive Oil or Nuts. N. Engl. J. Med..

[25] Fatima M., Muhammad Ali F., Ullah R. (2023). Popular Trend of Electronic Cigarettes and Their Adverse Effects on Oral Health. Cureus.

[26] Khudhur Z.O., Smail S.W., Awla H.K., Ahmed G.B., Khdhir Y.O., Amin K., Janson C. (2025). The effects of heavy smoking on oxidative stress, inflammatory biomarkers, vascular dysfunction, and hematological indices. Sci. Rep..

[27] Seo C.S., Chun J., Song K.H. (2025). Simultaneous Component Analysis of Akebia quinata Seeds (Lardizabalaceae) by Ultra-Performance Liquid Chromatography-Tandem Mass Spectrometry for Quality and Cytotoxicity Assessment. Plants.

[28] Wang Y., Feng Y., Li M., Yang M., Shi G., Xuan Z., Yin D., Xu F. (2022). Traditional Chinese Medicine in the Treatment of Chronic Kidney Diseases: Theories, Applications, and Mechanisms. Front. Pharmacol..

[29] Yu D., Huang N.N., Du X.W. (2023). Review of the Chemical Composition and Biological Activities of Essential Oils from Artemisia Argyi, Artemisia Princeps, and Artemisia Montana. Curr. Top. Med. Chem..

[30] Niu J., Li B., Zhang Q., Chen G., Papadaki A. (2025). Exploring the traditional Chinese diet and its association with health status-a systematic review. Nutr. Rev..

[31] Popkin B.M., Ng S.W. (2022). The nutrition transition to a stage of high obesity and noncommunicable disease prevalence dominated by ultra-processed foods is not inevitable. Obes. Rev..

[32] Mahanta B.P., Bora P.K., Kemprai P., Borah G., Lal M., Haldar S. (2021). Thermolabile essential oils, aromas and flavours: Degradation pathways, effect of thermal processing and alteration of sensory quality. Food Res. Int..

[33] Murphy S.E. (2017). Nicotine Metabolism and Smoking: Ethnic Differences in the Role of P450 2A6. Chem. Res. Toxicol..

[34] Butler K., Chenoweth M.J., El-Boraie A., Giratallah H., Kowalczyk W.J., Heishman S.J., Tyndale R.F., Le Foll B. (2021). Impact of CYP2A6 Activity on Nicotine Reinforcement and Cue-Reactivity in Daily Smokers. Nicotine. Tob. Res..

[35] Messina E.S., Tyndale R.F., Sellers E.M. (1997). A Major Role for CYP2A6 in Nicotine C-Oxidation by Human Liver Microsomes. J. Pharmacol. Exp. Ther..

[36] Murphy S.E., Raulinaitis V., Brown K.M. (2005). Nicotine 5’-oxidation and methyl oxidation by P450 2A enzymes. Drug Metab. Dispos..

[37] Siu E.C., Wildenauer D.B., Tyndale R.F. (2006). Nicotine self-administration in mice is associated with rates of nicotine inactivation by CYP2A5. Psychopharmacology.

[38] Raunio H., Pokela N., Puhakainen K., Rahnasto M., Mauriala T., Auriola S., Juvonen R.O. (2008). Nicotine metabolism and urinary elimination in mouse: In vitro and in vivo. Xenobiotica.

[39] Zhou X., Zhuo X., Xie F., Kluetzman K., Shu Y.Z., Humphreys W.G., Ding X. (2010). Role of CYP2A5 in the clearance of nicotine and cotinine: Insights from studies on a Cyp2a5-null mouse model. J. Pharmacol. Exp. Ther..

[40] Seo K.A., Kim H., Ku H.Y., Ahn H.J., Park S.J., Bae S.K., Shin J.G., Liu K.H. (2008). The monoterpenoids citral and geraniol are moderate inhibitors of CYP2B6 hydroxylase activity. Chem. Biol. Interact..

[41] Ilc T., Parage C., Boachon B., Navrot N., Werck-Reichhart D. (2016). Monoterpenol Oxidative Metabolism: Role in Plant Adaptation and Potential Applications. Front. Plant Sci..

[42] Duisken M., Sandner F., Blomeke B., Hollender J. (2005). Metabolism of 1,8-cineole by human cytochrome P450 enzymes: Identification of a new hydroxylated metabolite. Biochim. Biophys. Acta.

[43] Gyoubu K., Miyazawa M. (2007). In vitro metabolism of (-)-camphor using human liver microsomes and CYP2A6. Biol. Pharm. Bull..

[44] Subehan, Zaidi S.F., Kadota S., Tezuka Y. (2007). Inhibition on human liver cytochrome P450 3A4 by constituents of fennel (Foeniculum vulgare): Identification and characterization of a mechanism-based inactivator. J. Agric. Food Chem..

[45] Sang R., Jiang W., Zhang C., Yin R., Ouyang Z., Wei Y. (2025). Effect of food components on cytochrome P450 expression and activity. Hum. Nutr. Metab..

[46] Kim S.D., Morgan L., Hargreaves E., Zhang X., Jiang Z., Antenos M., Li B., Kirby G.M. (2021). Regulation of Cytochrome P450 2a5 by Artemisia capillaris and 6,7-Dimethylesculetin in Mouse Hepatocytes. Front. Pharmacol..

[47] Kondza M., Mandic M., Ivancic I., Vladimir-Knezevic S., Brizic I. (2023). Artemisia annua L. Extracts Irreversibly Inhibit the Activity of CYP2B6 and CYP3A4 Enzymes. Biomedicines.

[48] Pearson J., Gratale S.K., Ganz O., Erinoso O.A., Ohman-Strickland P., Wackowski O.A. (2025). Longitudinal relationship between relative harm perceptions, beliefs about organic and additive-free tobacco, and cigarette brand switching among Natural American Spirit, Camel and Marlboro cigarette smokers. Tob. Control..

[49] Faessel H.M., Obach R.S., Rollema H., Ravva P., Williams K.E., Burstein A.H. (2010). A review of the clinical pharmacokinetics and pharmacodynamics of varenicline for smoking cessation. Clin. Pharmacokinet..

[50] Jeong S.H., Newcombe D., Sheridan J., Tingle M. (2015). Pharmacokinetics of cytisine, an alpha4 beta2 nicotinic receptor partial agonist, in healthy smokers following a single dose. Drug Test. Anal..

[51] Lerman C., Schnoll R.A., Hawk L.W., Cinciripini P., George T.P., Wileyto E.P., Swan G.E., Benowitz N.L., Heitjan D.F., Tyndale R.F. (2015). Use of the nicotine metabolite ratio as a genetically informed biomarker of response to nicotine patch or varenicline for smoking cessation: A randomised, double-blind placebo-controlled trial. Lancet. Respir. Med..

[52] Khojasteh-Bakht S.C., Koenigs L.L., Peter R.M., Trager W.F., Nelson S.D. (1998). (R)-(+)-Menthofuran is a potent, mechanism-based inactivator of human liver cytochrome P450 2A6. Drug Metab. Dispos..

